# Roles of plasma leptin and resistin in novel subgroups of type 2 diabetes driven by cluster analysis

**DOI:** 10.1186/s12944-022-01623-z

**Published:** 2022-01-07

**Authors:** Xuemin Peng, Jiaojiao Huang, Huajie Zou, Bei Peng, Sanshan Xia, Kun Dong, Nan Sun, Jing Tao, Yan Yang

**Affiliations:** 1 Department of Endocrinology, Tongji Hospital, Tongji Medical College, Huazhong University of Science and Technology, Wuhan, 430030 Hubei China; 2 Department of Endocrinology, TaiKang Tongji (Wuhan) Hospital, Wuhan, 430030 Hubei China; 3 Branch of National Clinical Research Center for Metabolic Diseases, Wuhan, Hubei China

**Keywords:** Type 2 diabetes mellitus, Leptin, Resistin, Cluster analysis, Novel subgroups

## Abstract

**Background:**

A novel classification has been introduced to promote precision medicine in diabetes. The current study aimed to investigate the relationship between leptin and resistin levels with novel refined subgroups in patients with type 2 diabetes mellitus (T2DM).

**Methods:**

The k-means analysis was conducted to cluster 541 T2DM patients into the following four subgroups: mild obesity-related diabetes (MOD), severe insulin-deficient diabetes (SIDD), severe insulin-resistant diabetes (SIRD) and mild age-related diabetes (MARD). Individuals meeting the exclusion criteria were eliminated, the data for 285 patients were analyzed. Characteristics were determined using various clinical parameters. Both the leptin and resistin levels were determined using enzyme-linked immunosorbent assay.

**Results:**

The highest levels of plasma leptin were in the MOD group with relatively lower levels in the SIDD and SIRD groups (*P* < 0.001). The SIRD group had a higher resistin concentration than the MARD group (*P* = 0.024) while no statistical significance in resistin levels was found between the SIDD and MOD groups. Logistic regression demonstrated that plasma resistin was associated with a higher risk of diabetic nephropathy (odds ratios (OR) = 2.255, *P* = 0.001). According to receiver operating characteristic (ROC) curves, the area under the curve (AUC) of resistin (0.748, 95% CI 0.610–0.887) was significantly greater than that of HOMA2-IR (0.447, 95% CI 0.280–0.614) (*P* < 0.05) for diabetic nephropathy in the SIRD group.

**Conclusions:**

Leptin levels were different in four subgroups of T2DM and were highest in the MOD group. Resistin was elevated in the SIRD group and was closely related to diabetic nephropathy.

**Supplementary Information:**

The online version contains supplementary material available at 10.1186/s12944-022-01623-z.

## Background

In China, the overall prevalence of diabetes mellitus reached approximately 10.9% in 2013 and has rapidly increased in recent years [1]. Overall, available treatments and management strategies are of limited efficacy and cannot slow the progression of diabetes. One possible reason is that type 2 diabetes mellitus (T2DM) has high heterogeneity and diabetes classification is too simple. To address this issue, in 2018, Ahlqvist et al. firstly defined five diabetic subgroups according to six different variables (glutamate decarboxylase antibodies (GADA), age at diagnosis, body mass index (BMI), hemoglobin A1c (HbA1c), homeostatic model assessment 2 estimates of β-cell function (HOMA2-B) and insulin resistance (HOMA2-IR)) to provide more individual and precise strategies. Among the variables, the HOMA2-IR and HOMA2-B are indexes of insulin resistance and insulin secretory function respectively [2]. These subgroups were the following: severe autoimmune diabetes (SAID) group, mild obesity-related diabetes (MOD) group, severe insulin-deficient diabetes (SIDD) group, severe insulin-resistant diabetes (SIRD) group and mild age-related diabetes group (MARD) [3]. With GADA as a binary variable, the GADA-positive diabetic patients were classified into the SAID group. In Zou’s study, the diabetic patients who are GADA negative (namely T2DM patients) were stratified into four subgroups (MOD, SIDD, SIRD, MARD) using five variables (age at diagnosis, BMI, HbA1c, HOMA2-B and HOMA2-IR) using Chinese populations and obtained similar conclusions [4]. In the current study, T2DM patients were also stratified into the above four groups using the same methods. According to Ahlqvist’s study, each group has its distinctive characteristics. Patients with obesity (higher BMI) but no insulin resistance are classified into the MOD group. The SIDD group is characterized by insulin-deficient diabetes and GADA negativity. The SIRD group has a higher risk of diabetic nephropathy and an evidently high HOMA2-IR index. Older diabetic patients with modest metabolic disorders are defined as the MARD group [3].

Adipose tissue is a key endocrine organ that communicates with the brain, muscle, liver, and pancreas, thereby, maintaining energy homeostasis. Secretion of adipokines including leptin and resistin is altered in adipose tissue dysfunction and may contribute to diabetes, which may provide promising novel pharmacological treatment strategies for diabetes [5]. Among the adipokines, leptin and resistin were important that influenced both insulin sensitivity and inflammation, which were closely linked to the development of T2DM [6].

Leptin, a peptide of 167 amino acids [7], acts as an essential hormone to regulate energy balance [8]. People with leptin deficiency exhibit increased food intake, adipose stores and weight gain. Leptin can also regulate glucose homeostasis and insulin function. For example, leptin-deficient rodents and humans, which are generally characterized by obesity, impaired glucose tolerance, insulin resistance as well as hyperinsulinemia, can be normalized by leptin therapy [9]. However, leptin administration has been unsuccessful in improving glucose homeostasis in many clinical trials [10, 11]. In general, analysis of the association between leptin and novel subgroups of diabetes is valuable for leptin’s clinical applications.

Resistin, a 108-amino acid polypeptide derived from peripheral blood mononuclear cells in humans, is different from its secretion in rodents as an adipocyte-derived protein [12]. The physiological role of resistin is to promote immune and proinflammatory processes [13]. A hyperresistinemic state enhances the incidence of coronary heart disease [14], liver disease [15] and kidney failure [16]. In rodents, positive associations are found between resistin expressed in white adipocytes with BMI and insulin resistance. However, the role of resistin in diabetic patients is still controversial. Some studies reported that resistin served as an important regulator of contributing to insulin resistance [17, 18] while consistent results were not reported in another study [19]. The four novel subgroups driven by cluster analysis will unravel the role of resistin in diabetes.

The current work was to illuminate the relation between leptin and resistin levels with novel T2DM subgroups. A refined classification of T2DM may deepen the understanding of the characteristics of leptin and resistin and help to precisely treat T2DM.

## Materials and methods

### Study population

From 2017 to 2019, 541 consecutive T2DM inpatients from Tongji Hospital were included in the current study. The inclusion criteria were the following: [1] adults (over 18 years old) and [2] meeting the diagnostic criteria [20], i.e. fasting glucose ≥7.0 mmol/L, random glucose ≥11.1 mmol/L, or 2-h plasma glucose ≥11.1 mmol/L. T2DM patients were newly-diagnosed through the classical oral glucose tolerance test after admission or confirmed according to the medical history. Diabetic nephropathy is diagnosed as an increase in urinary albumin excretion (≥ 30 mg/24 h) or an increase in the albumin-to-creatinine ratio (> 2.5 mg/mmol in males and over 3.5 mg/mmol in females) and a reduction in renal function as reflected by a decreased glomerular filtration rate (GFR) [21]. Cluster analysis was conducted on these 541 patients as follows: MOD, SIDD, SIRD and MARD groups. After eliminating the individuals meeting the exclusion criteria similar to the Ahlqvist’s study [1, 3] missing clinical data [2]; secondary diabetes [3]; extreme outliners, leptin and resistin levels were finally analyzed from 285 patients. The sample size was sufficient to detect the clustering effect according to the preliminary experiment and analysis.

The ethics committee of Tongji Hospital approved the study design (IRB ID: TJ-C20160206). This study complied with the Declaration of Helsinki provisions and oral informed consent was obtained from the patients.

### Measurements

Leptin and resistin levels were examined through a Human Quantikine enzyme-linked immunosorbent assay (R&D Systems Inc., Minneapolis, Minnesota, United States, Cat# DLP00, Ca# DRSN00) in duplicate in line with the manufacturer’s instructions. Fasting plasma glucose (FPG), C-peptide and HbA1c levels, alanine transaminase (ALT), glutamic oxalacetic transaminase (AST), total cholesterol (TC), blood urea nitrogen (BUN), serum creatinine (SCr) as well as eGFR were measured. A repeated sample was detected to verify these results. BMI, HOMA2-B and HOMA2-IR were confirmed according to the calculator in a previous study [22].

### Cluster analysis

Given that GADA-positive diabetic patients are generally diagnosed before capture by screening for diabetes complications and that the prevalence of GADA-positive T2DM is only 5.9% in China [4], GADA was not tested as detecting GADA-positive diabetes was difficult. The data were clustered into four groups (MOD, SIDD, SIRD, and MARD) by k-means analysis based on age at diagnosis, BMI, HbA1c, HOMA2-B and HOMA2-IR, which was similar to Zou et al. [4]. To decrease stratification from sex-associated differences and provide separate cohorts to validate results, data for men and women were clustered separately. Then k-means clustering was conducted by a k value of four through the k-means runs function (runs = 100) in the TensorFlow 2.0. Finally, the four cluster results in 3D were visualized through T-distributed stochastic neighbor embedding [23].

### Statistical analysis

Mean ± standard deviation was applied for continuous variables in the normal distribution, otherwise the interquartile range was used. Numbers (percentages) presented categorical variables. Characteristics of participants were compared using the chi-squared test for categorical variables. Differences between subgroups were evaluated by ANOVA and by post hoc test between groups (Bonferroni correction) for normally distributed variables. The Kruskal-Wallis test along with Bonferroni correction was adopted for skewed distributions. Logistic regression was also adopted to find the relationship between adipocytokines and diabetic complications.

The optimal cutoff point, sensitivity and specificity were assessed by receiver operating characteristic (ROC) curves. The measurement of predictive values for diabetic nephropathy relied on the area under the curve (AUC) values.

SPSS (Version 24.0; Chicago, IL, USA) was applied in all statistical analyses employing two-tailed tests. A significant difference was considered if *P* < 0.05 in all statistical tests.

## Results

### Characteristics of the study population

**Fig. 1:**
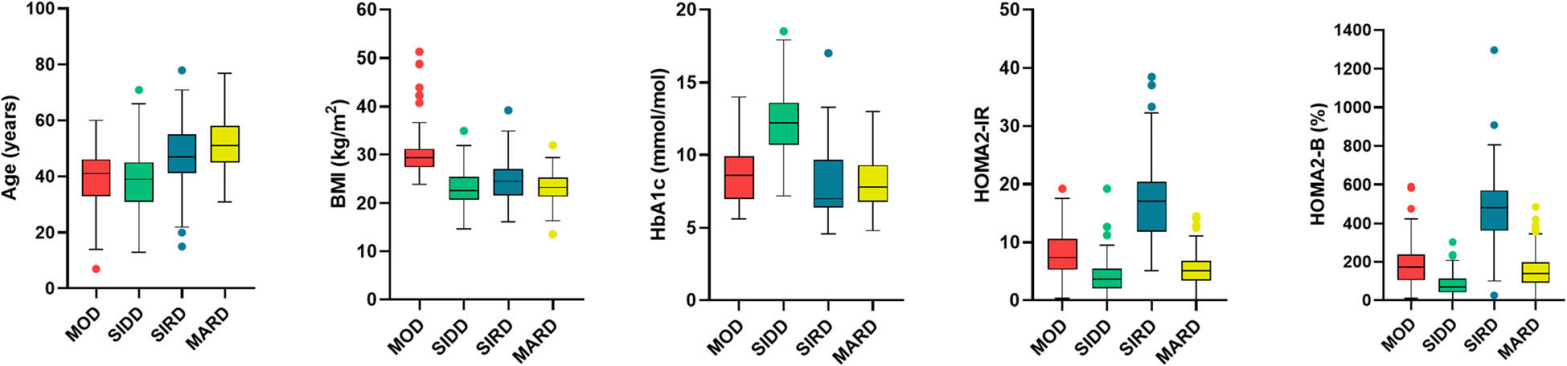
Cluster characteristics in the diabetic participants. Bar chart showing the characteristics of the five variables. Distributions of onset age, BMI, HbA1c, HOMA2-IR and HOM2A-B in the 541 diabetic participants for each cluster. k-means clustering was performed, and the characteristics of the clusters were compared, similar to a previous study. MOD, mild obesity-related diabetes; SIDD, severe insulin-deficient diabetes; SIRD, severe insulin-resistant diabetes; MARD, mild age-related diabetes; BMI, body mass index; HbA1c, hemoglobin; HOMA2-IR, homeostasis model assessment of insulin resistance; HOMA2-B, homeostasis model assessment of beta-cell function

A total of 285 patients was analyzed after applying the exclusion criteria (Table 1). The overall cluster characteristics of the five variables were described in Fig. 1 in line with Ahlqvist’s study (*P* values for five variables (age at diagnosis, BMI, HbA1c, HOMA2-B and HOMA2-IR) within the different subgroups (MOD, SIDD, SIRD, MARD) < 0.001). Overall, the results showed that the levels of leptin, resistin, ALT, AST, SCr and eGFR differed significantly among the novel subgroups (all *P* < 0.05) (Table 1).

**Table 1:** Clinical and metabolic parameters for included subjects in novel subgroups of 285 adult diabetes

Characteristics	MOD	SIDD	SIRD	MARD	*P* value
N (%)	50 (17.5)	71 (24.8)	48 (16.8)	116 (40.9)	
Age (years)	39.06 ± 10.07	37.66 ± 11.68	47.56 ± 10.90	51.98 ± 10.05	< 0.001
Male(%)	31 (62.0)	49 (69.0)	35 (72.9)	65 (56.0)	0.135
BMI (kg/m2)	30.80 ± 4.86	23.26 ± 3.45	24.70 ± 3.86	23.32 ± 2.94	< 0.001
HbA1c	8.40	11.80	7.50	7.85	< 0.001
(%)	(6.90–9.90)	(10.46–13.60)	(6.43–9.78)	(6.73–9.25)	
HOMA2-IR	7.78	3.66	17.24	4.59	< 0.001
	(5.04–12.50)	(1.97–5.62)	(11.94–20.31)	(2.98–6.85)	
HOMA2-B	178.20	70.70	461.80	139.50	< 0.001
	(92.58–266.25)	(48.90–111.40)	(350.88–576.25))	(89.05–196.98)	
‘Leptin	7.71	3.92	4.48	3.91	< 0.001
(ng/mL)	(4.22–15.20)	(2.38–7.19)	(2.74–7.81)	(2.44–7.25)	
Resistin	9.22	8.00	12.05	6.36	0.014
(ng/mL)	(6.29–13.20)	(4.80–15.33)	(5.16–18.49)	(3.85–11.40)	
ALT	25.50	16.00	20.50	19.00	0.035
(U/L)	(15.75–50.25)	(12.25–25.75)	(13.75–29.25)	(14.00–31.00)	
AST	18.50	16.00	20.00	19.00	0.001
(U/L)	(15.00–35.50)	(12.50–21.50)	(16.75–27.25)	(17.00–26.00)	
TC	4.05	4.32	4.00	3.98	0.550
(mmol/L)	(3.46–4.78)	(3.58–4.87)	(3.38–4.76)	(3.00–4.83)	
BUN	5.45	6.00	6.44	5.80	0.101
(mmol/L)	(4.38–7.16)	(4.70–7.68)	(5.16–8.20)	(4.60–7.00)	
SCr	69.00	67.00	95.00	70.00	< 0.001
(μmol/L)	(55.50–83.00)	(50.00–77.00)	(64.75–132.75)	(56.00–88.00)	
eGFR	102.80	109.60	76.60	92.00	< 0.001
(mL/min/1.73m^2^)	(87.30–115.05)	(97.03–122.23)	(46.90–99.70)	(75.30–102.40)	

*MOD* mild obesity-related diabetes, *SIDD* severe insulin-deficient diabetes, *SIRD* severe insulin-resistant diabetes, *MARD* mild age-related diabetes, *BMI* body-mass index, *HbA1c* hemoglobin, *HOMA-IR* homeostasis model assessment of insulin resistance, *HOMA-B* homeostasis model assessment of beta cell function, *ALT* alanine transaminase, *AST* aspartate transaminase, *TC* total cholesterol, *BUN* blood urea nitrogen, *SCr* Serum Creatinine, *eGFR* glomerular filtration rate

### Comparisons of leptin and resistin in four novel subgroups

**Fig. 2:**
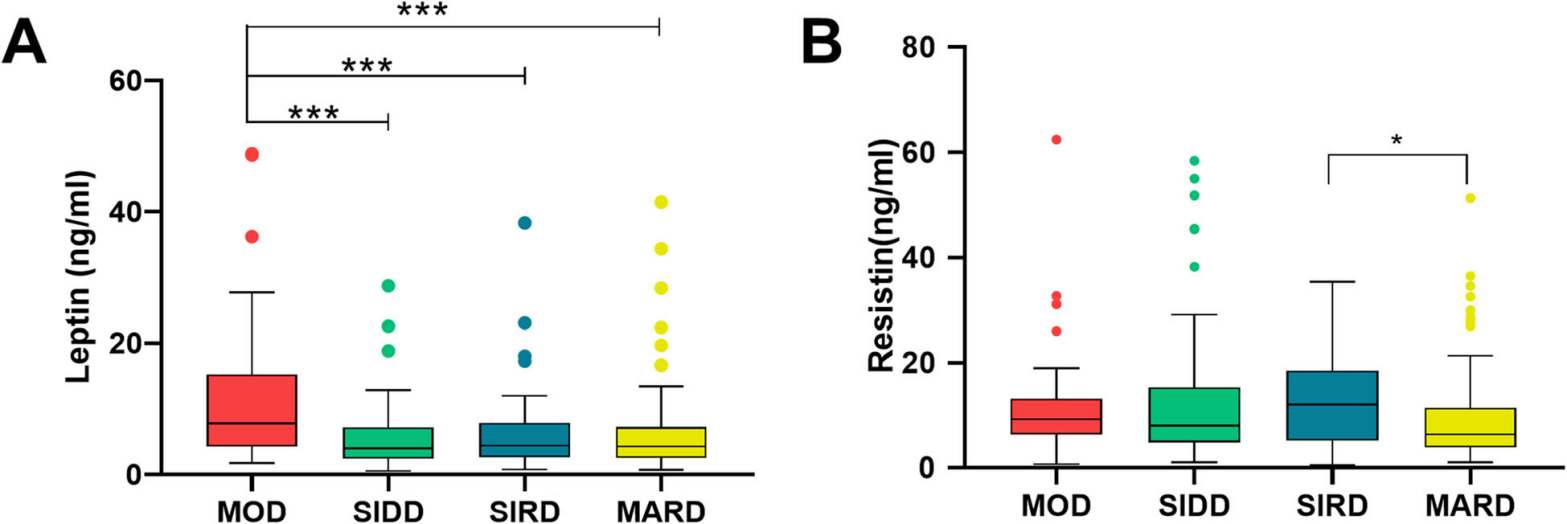
Comparisons of leptin and resistin in four novel subgroups. Pairwise comparisons of leptin and resistin among the four subgroups. As shown in Fig. 2A, leptin levels were the highest in the MOD group (*P* < 0.001). There was no significant difference in leptin levels in the SIDD, SIRD and MARD groups. (*P* > 0.05). As shown in Fig. 2B, resistin levels in the SIRD group were higher than those in the MARD group (*P* = 0.024), whereas there were almost no differences between the SIDD and MOD groups (*P* > 0.05). * indicates *P* < 0.05, ** indicates *P* < 0.01, *** indicates *P* < 0.001

After pairwise comparisons among the four subgroups, the highest leptin levels were detected in the MOD group (*P* < 0.001, Table 1, Fig. 2A). Alternatively, leptin levels were relatively lower in the SIDD and SIRD groups as compared to the MOD group. No statistical significance in leptin levels was found among the other three groups (*P* > 0.05, Fig. 2A).

A higher concentration of resistin was observed in the SIRD group than in the MARD group (*P* = 0.024, Table 1, Fig. 2B). However, no statistical significance in resistin levels was found between the SIRD, SIDD and MOD groups (*P* > 0.05) (Fig. 2B).

### Leptin was shown to be associated with BMI, whereas resistin was related to renal function

To further evaluate which factors were relevant in the relationship between diabetes and leptin, correlation analysis of leptin with five variables and clinical indexes was conducted (Table 2). Positive associations were observed between leptin levels with BMI (r = 0.520, *P* < 0.001) and HOMA2-IR (r = 0.130, *P* = 0.028). Although the association between leptin levels with HOMA2-IR disappeared when BMI was adjusted (r = 0.030, *P* = 0.614), BMI remained statistically significant after multiple correction (r = 0.508, *P* < 0.001). Additionally, similar results for each subgroup were obtained in a correlation analysis (Supplemental Tables 1–4).

**Table 2:** Correlations analysis between adipokines levels and clinical parameters in study participants

	Leptin		Resistin	
	*r*	*P* value	*r*	*P* value
Age	−0.097	0.104	−0.043	0.468
BMI	0.520	< 0.001	0.041	0.487
HbA1c	−0.007	0.900	0.069	0.245
HOMA-IR	0.130	0.028^a^	0.063	0.289
HOMA-B	0.078	0.186	0.082	0.169
ALT	0.059	0.324	0.073	0.222
AST	0.032	0.569	0.095	0.112
TC	0.095	0.112	0.038	0.527
BUN	0.038	0.529	0.260	< 0.001
SCr	0.032	0.589	0.335	< 0.001
eGFR	−0.086	0.149	−0.339	< 0.001
Albumin-to-creatinine ratio	0.041	0.566	0.380	< 0.001

^a^After adjustment BMI, there was no significant association between Leptin and HOMA-IR (r = 0.030, *P* = 0.614)

Correlation analysis was also conducted to identify factors involved in the association between resistin with the novel subgroups (Table 2). Resistin had a positive association with some renal function indicators such as BUN (r = 0.260, *P* < 0.001), SCr (r = 0.335, *P* < 0.001) and albumin-to-creatinine ratio (r = 0.380, *P* < 0.001) and had a negative association with eGFR (r = − 0.339, *P <* 0.001). Similar results were also found in all four subgroups (Supplemental Tables 1–4). Conversely, insulin resistance was not correlated to resistin (Table 2, Supplemental Tables 1–4).

### Resistin was assessed to be more closely associated with diabetic nephropathy than HOMA2-IR

Patients in the SIRD group had a more than 2-fold risk of diabetic kidney disease as the sex and age of onset were adjusted (OR = 2.332, 95% CI 1.148–4.735, *P* = 0.019, Table 3) compared with the MARD group in this study, which was similar to Ahlqvist’s study. Risk factors for diabetic nephropathy as well as comparisons between resistin and insulin resistance were investigated by logistic regression analysis.

**Table 3:** Logistic regression analysis of risk factors for kidney complications in diabetic participants

		OR (95%CI) ^a^	*P* ^a^	OR (95%CI) ^b^	*P* ^b^
Subgroups					
	MOD	0.933 (0.472–1.844)	0.841	1.079 (0.509–2.288)	0.842
	SIDD	1.656 (0.912–3.005)	0.097	1.880 (0.947–3.731)	0.071
	SIRD	2.130 (1.075–4.221)	0.030	2.332 (1.148–4.735)	0.019
	MARD	Ref		Ref	
Resistin	By median ^c^				
	<Median	Ref		Ref	
	≥Median	2.262 (1.405–3.641)	0.001	2.255 (1.388–3.663)	0.001
	By quartile ^d^				
	Q1	Ref		Ref	
	Q2	1.391 (0.698–2.773)		1.418 (0.706–2.848)	
	Q3	1.629 (0.826–3.211)		1.704 (0.859–3.383)	
	Q4	4.087 (2.031–8.233)	0.001	4.010 (1.976–8.135)	0.001
HOMA-IR	By median				
	<Median	Ref		Ref	
	≥Median	1.656 (1.035–2.650)	0.036	1.620 (1.005–2.614)	0.048
	By quartile				
	Q1	Ref		Ref	
	Q2	1.197 (0.607–2.361)		1.198 (0.603–2.381)	
	Q3	1.519 (0.780–2.956)		1.430 (0.726–2.817)	
	Q4	2.184 (1.112–4.287)	0.121	2.228 (1.122–4.423)	0.120

^a^ Logistic regression was unadjusted

^b^ Logistic regression was adjusted for gender and age of onset

^c^ The median of all subjects were used as cutoffs when creating groups

^d^ The quartile values of all subjects were used as cutoffs when creating groups

Q1, first quartile; Q2, second quartile; Q3, third quartile; Q4, fourth quartile

When subjects were categorized into two comparable groups by median value, there was a nearly 2.3-fold and 1.6-fold increased risk of renal complications among the groups with higher resistin levels (odds ratios (OR) = 2.255 after adjustment, 95% confidence interval (CI) = 1.388–3.663, *P* = 0.001) and HOMA-IR (adjusted OR = 1.620, 95% CI 1.005–2.614, *P* = 0.048) respectively (Table 3).

When quartile values of risk factors were used as cutoff points, the highest resistin quartile showed a four-fold risk of kidney injury compared to the first resistin quartile (adjusted OR = 4.010, 95% CI 1.976–8.135, *P* = 0.001, Table 3). However, no significant trend of HOMA2-IR was found (*P* for trend = 0.120). Therefore, resistin may be more closely associated with diabetic nephropathy than HOMA2-IR.

Comparisons of resistin and HOMA2-IR in each subgroup were shown in Supplemental Tables 5–8. The results for the SIRD and MARD groups were similar to the conclusions above and there was no statistical significance between resistin and renal complications in the MOD and SIDD groups.

### Resistin showed closer links with diabetic nephropathy than insulin resistance in the SIRD group

**Fig. 3:**
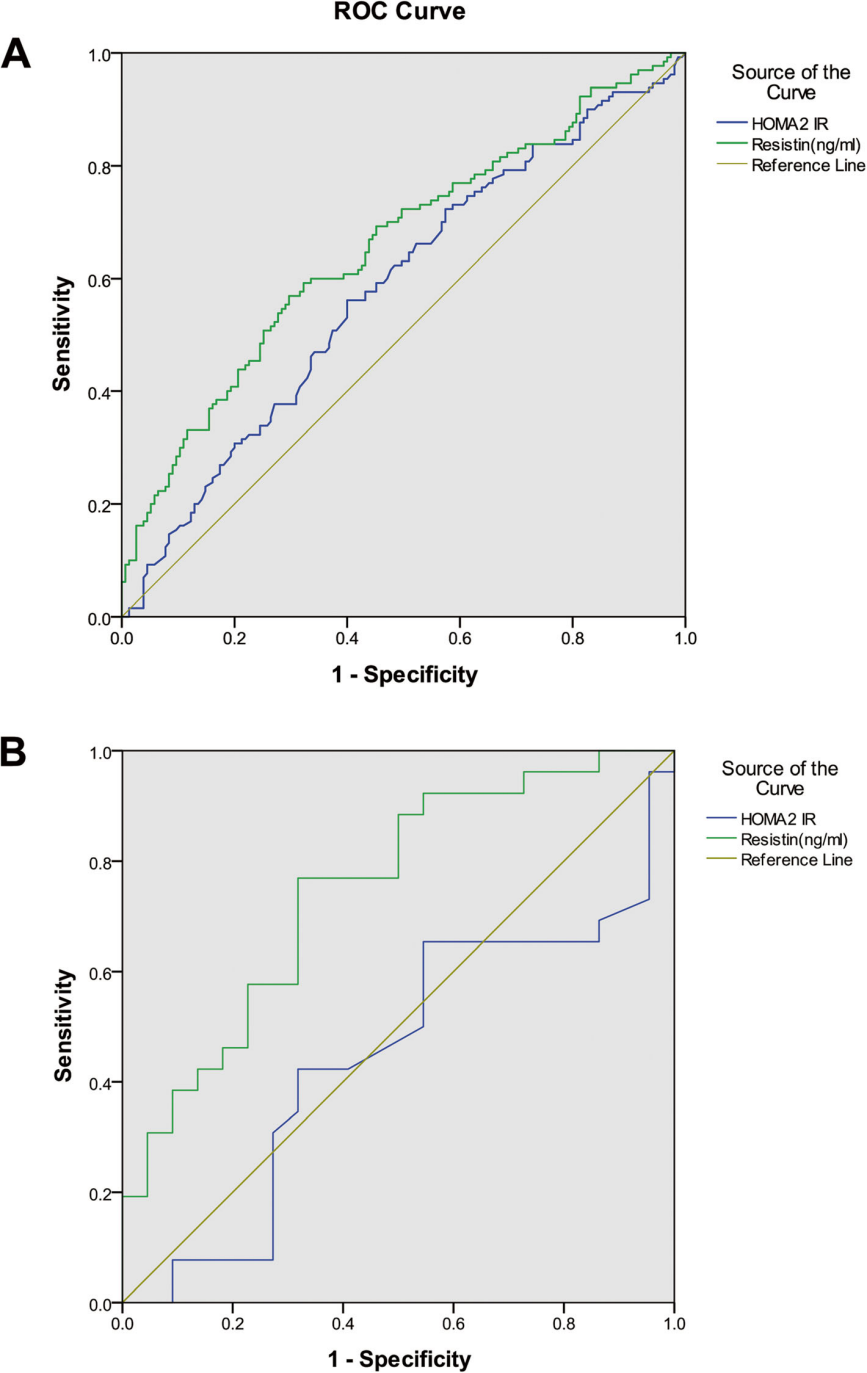
Receiver operating characteristic (ROC) curve analysis of resistin and HOMA2-IR for diabetic nephropathy. ROC curves and corresponding areas under the curve (AUCs) for diabetic nephropathy in a total of 285 patients (Fig. 3A) and in the SIRD subgroup of 48 patients (Fig. 3B). AUCs of resistin and HOMA2-IR were 0.656 (95% CI 0.592–0.720) and 0.581 (95% CI 0.515–0.648), respectively. In the SIRD group, AUCs of resistin and HOMA2-IR were 0.748 (95% CI 0.610–0.887) and 0.447 (95% CI 0.280–0.614), respectively

ROC plots were generated to assess the predictive value of resistin and HOMA2-IR for diabetic nephropathy. The AUCs were 0.656 (95% CI 0.592–0.720) for resistin and 0.581 (95% CI 0.515–0.648) for HOMA2-IR in all participants (Fig. 3A). The sensitivity and specificity of resistin (sensitivity 56.9%, specificity 70.3%) and HOMA2-IR (sensitivity 56.2%, specificity 60.0%) were also evaluated. Although the AUCs in the two factors did not exhibit significant differences (*P* > 0.05), the AUC for resistin was larger than that for HOMA2-IR (Fig. 3A).

In the SIRD group, the AUC for HOMA2-IR (0.447, 95% CI 0.280–0.614) was also significantly lower than that of resistin (0.748, 95% CI 0.610–0.887) as depicted in Fig. 3B (*P* = 0.007). The AUC for resistin approached the upper-left corner in the coordinate axis with 76.9 and 68.2% sensitivity and specificity, respectively. Comparisons of the other three groups were shown in Supplemental Fig. 1.

## Discussion

In the present study, the novel T2DM classification was based on five variables, which was confirmed to benefit the diagnosis of diabetic complications and personalized regimens. The clustering efficiency of this study was in line with that of Ahlqvist’s study, and comparisons of leptin and resistin were conducted among the novel subgroups.

The leptin receptor (LepRb) is primarily expressed in the arcuate nucleus (ARC) and ventromedial nucleus of the hypothalamus [24]. Agouti-related protein neurons located in the ARC are critical for regulating appetite, which is assumed to be responsible for obesity [25]. As expected, leptin levels were the highest in the MOD subgroup and were positively associated with BMI in the analysis (Fig. 2, Table 2). Other factors such as HbA1c and insulin homeostasis index were all unrelated to leptin (Table 2). Relatively lower levels of leptin were present in the MARD, SIDD and SIRD groups (Fig. 2).

Leptin is a potential therapeutic candidate for T2DM due to its attractive advantage in lowering glucose levels. Indeed, leptin can independently lower blood glucose levels particularly in hyperglycemic models of insulin deficiency (rodents with streptozotocin injection or insulin knockout) [24]. Numerous observations suggest that these beneficial effects are independent of reduction in body weight [24]. Distinctive mechanisms of lowering diabetes such as the increased sympathetic nerve activity in peripheral tissue and the suppression of the hypothalamic-pituitary-adrenal axis pathway through the central nervous system play an important role. However, the clinical application of leptin is limited to leptin-deficient individuals. Leptin failed to improve insulin sensitivity in T2DM patients with obesity [10] due to hyperleptinemia and leptin resistance [26, 27]. More importantly, these trials were conducted in an unselected population with obesity [8, 28]. The proposed novel classification of diabetes may contribute in selecting a suitable population for leptin therapy. First, the SIDD and SIRD groups had relatively lower leptin levels as compared to the MOD group, and patients who may progress to leptin resistance because of higher leptin levels had been filtered. Moreover, leptin administration has achieved promising results in insulin-deficient rodent models, which indicated leptin could reverse hyperglycemia independent of insulin [29]. Leptin’s role in enhancing insulin sensitivity [30] and improving insulin resistance in lipodystrophy and diabetic models has also been reported [31, 32]. Given that SIDD and SIRD groups with low leptin levels featured insulin deficiency and insulin resistance, respectively, individuals classified into these two groups may benefit from leptin replacement therapy.

Resistin was initially described as an adipocyte-derived protein with a metabolic effect on insulin resistance in mice [12]. However, there are many debates on the association between resistin and insulin resistance in human studies [33]. This issue was discussed from the perspective of novel T2DM classifications. Correlation analyses between the resistin and insulin resistance were conducted in the total group and the four subgroups. However, no significant relationship was observed between resistin with insulin resistance, even in the SIRD group (Table 2, Supplementary Table 3).

Recently, the role of resistin in inflammation in humans has been widely recognized [33]. The expression of several proinflammatory cytokines in peripheral blood mononuclear cells, like TNF-α, interleukin-6, C-reactive protein or monocyte chemoattractant protein-1, is upregulated by resistin [34]. Multiple reports highlighted that resistin is a biomarker of inflammation in diabetes as well as in many other inflammatory conditions, like sepsis, inflammatory bowel disease as well as rheumatoid arthritis [35, 36]. Thus, the SIRD group with high resistin levels may have a relatively high inflammatory state in addition to insulin resistance.

At the same time, resistin was shown to be related to diabetic nephropathy in the logistic regression analysis in the results (Table 3). Compared with insulin resistance, resistin had a closer relationship with renal complications in the SIRD group based on ROC curve analysis (Fig. 3B). The inflammation pathway mediated by resistin is considered responsible for this result, which has been demonstrated in kidney injury [37]. Resistin decreases neutrophil chemotaxis and oxidative stress via inhibition of the PI3K signaling pathway to increase kidney damage in vitro [38]. It is possible that in the SIRD group, resistin participates in a more important mechanism to promote diabetic nephropathy attributable to its proinflammatory potential. Insulin resistance has been widely accepted in T2DM administration. Many medicines for improving insulin resistance have been generalized for patients with diabetes. However, there are few regimens for alleviating inflammation. Resistin-related pathways should garner attention especially in the treatment of diabetic nephropathy which may be more relevant to the progression of kidney injury in the SIRD group.

This study firstly analyzed the relationship between leptin and resistin in T2DM according to novel subgroups, providing promising prospects for precision medicine involving leptin or resistin in diabetes. Previous studies have suggested leptin might help in the increased insulin sensitivity as well as improved insulin resistance [29, 30] while such benefits are absent in diabetic patients with obesity due to leptin resistance [10]. The SIDD and SIRD groups with low leptin levels and characterized by insulin deficiency or insulin resistance under the precise stratification may be appropriate for leptin therapy. Additionally, many studies have linked high resistin levels with diabetic nephropathy while few studies have linked them to novel subgroups of T2DM patients [22, 39, 40]. Although previous publications have explored the relationship between resistin and diabetic nephropathy after stratifying patients by BMI or non-alcoholic fatty liver [22, 39], the present study provides more precise results based on five variables rather than merely one indicator. The findings indicated that resistin might be an effective predictor for diabetic nephropathy in the SIRD group.

### Study strengths and limitations

The study has several strengths. For one, this study firstly analyzed the role of leptin and resistin in T2DM according to the proposed novel subgroups. Second, leptin levels varied among T2DM subgroups which might provide precise applications for leptin therapy. Finally, resistin levels were higher in the SIRD group and more closely related to diabetic nephropathy than insulin resistance. Resistin-related mechanisms should be of great concern during the treatment of diabetic nephropathy. There are also several limitations of this research. First, this study was conducted only in Asian populations and the sample size was relatively small. Nevertheless, the prevalence of cardiovascular risk factors in one population is similar to that of other large contemporary trials including several other ethnicities [41, 42], which might potentially support the generalizability of the results of the current study. Larger samples are needed to further confirm the results. Second, this study did not administer leptin therapy to the SIDD and SIRD populations and did not measure other classic inflammatory factors to identify the role of resistin in the latter group. More work is needed to investigate the exact mechanism in future studies.

## Conclusions

In conclusion, the findings showed high leptin levels in the MOD group were associated with BMI. In contrast, the SIDD and SIRD groups with relatively lower leptin levels might be appropriate for leptin therapy. The SIRD group had high levels of resistin, and resistin may serve as a promising predictor for diabetic nephropathy in this group.

## Supplementary Information

**Additional file 1: Supplementary Tables 1–4.** Correlations Analysis Between Adipokines Levels and Clinical Parameters in the MOD, SIDD, SIRD and MARD subgroups.

**Additional file 2: Supplementary Tables 5–8.** Logistic regression analysis of risk factors for renal complications in the MOD, SIDD, SIRD and MARD subgroups.

**Additional file 3: Supplementary Fig. 1.** Receiver operating characteristic (ROC) curve analysis of resistin and HOMA2-IR for diabetic nephropathy in the other three subgroups (MOD, SIDD and MARD)

## Data Availability

The data used and/or analyzed during the current study are available from the corresponding author on reasonable request.

## Abbreviations

T2DM: type 2 diabetes mellitus
GADA: glutamate decarboxylase antibodies
BMI: body mass index
HbA1c: hemoglobin A1c
HOMA2-B: homeostatic model assessment 2 estimates of β-cell function
HOMA2-IR: homeostatic model assessment 2 estimates of insulin resistance
SAID: severe autoimmune diabetes
MOD: mild obesity-related diabetes
SIDD: severe insulin-deficient diabetes
SIRD: severe insulin-resistant diabetes
MARD: mild age-related diabetes
UAE: urinary albumin excretion
eGFR: decreased glomerular filtration rate
ALT: alanine transaminase
AST: aspartate transaminase
TC: total cholesterol
BUN: blood urea nitrogen
SCr: serum creatinine
CIs: confidence intervals
OR: odds ratios
ROC: receiver operating characteristic
AUC: area under the curve
ARC: arcuate nucleus
