# Supplementary material for: Roles of plasma leptin and resistin in novel subgroups of type 2 diabetes driven by cluster analysis
Source: Lipids Health Dis. 2022 Jan 7;21:7. doi: 10.1186/s12944-022-01623-z (PMC8742314; doi:10.1186/s12944-022-01623-z)
Supplement: Supplementary file 1 — Additional file 1: Supplementary Tables 1–4. Correlations Analysis Between Adipokines Levels and Clinical Parameters in the MOD, SIDD, SIRD and MARD subgroups. [file 12944_2022_1623_MOESM1_ESM.docx]

**Supplementary tables 1-4**

**Sup Table 1 Correlations Analysis Between Adipokines Levels and Clinical Parameters in the MOD group**

|  | Leptin | | Resistin | |
| --- | --- | --- | --- | --- |
|  | *r* | *P* value | *r* | *P* value |
| Age(years) | -0.296 | 0.037 | -0.024 | 0.867 |
| BMI(kg/m^2^) | 0.584 | <0.001 | 0.351 | 0.012 |
| HbA1c(%) | 0.320 | 0.023 | 0.183 | 0.204 |
| HOMA-IR | 0.183 | 0.202 | 0.096 | 0.509 |
| HOMA-B | 0.215 | 0.134 | 0.075 | 0.603 |
| ALT(U/L) | 0.178 | 0.217 | 0.039 | 0.786 |
| AST(U/L) | 0.149 | 0.303 | 0.017 | 0.905 |
| TC(mmol/L) | 0.177 | 0.220 | 0.209 | 0.145 |
| BUN(mmol/L) | 0.195 | 0.175 | 0.533 | <0.001 |
| SCr(μmol/L) | 0.112 | 0.438 | 0.301 | 0.033 |
| eGFR(mL/min/1.73m^2^) | -0.097 | 0.508 | -0.256 | 0.076 |
| Albumin-to-creatinine ratio | 0.427 | 0.013 | 0.698 | <0.001 |

**Sup Table 2 Correlations Analysis Between Adipokines Levels and Clinical Parameters in the SIDD group**

|  | Leptin | | Resistin | |
| --- | --- | --- | --- | --- |
|  | *r* | *P* value | *r* | *P* value |
| Age(years) | -0.126 | 0.294 | -0.041 | 0.732 |
| BMI(kg/m^2^) | 0.311 | 0.008 | -0.042 | 0.727 |
| HbA1c(%) | -0.025 | 0.839 | -0.094 | 0.436 |
| HOMA-IR | -0.070 | 0.561 | 0.056 | 0.643 |
| HOMA-B | 0.015 | 0.904 | -0.060 | 0.620 |
| ALT(U/L) | -0.098 | 0.426 | 0.281 | 0.020 |
| AST(U/L) | -0.074 | 0.548 | 0.307 | 0.010 |
| TC(mmol/L) | -0.035 | 0.771 | -0.064 | 0.594 |
| BUN(mmol/L) | 0.190 | 0.112 | 0.126 | 0.297 |
| SCr(μmol/L) | 0.142 | 0.236 | 0.264 | 0.026 |
| eGFR(mL/min/1.73m^2^) | -0.109 | 0.369 | -0.302 | 0.011 |
| Albumin-to-creatinine ratio | -0.044 | 0.751 | 0.568 | <0.001 |

**Sup Table 3 Correlations Analysis Between Adipokines Levels and Clinical Parameters in the SIRD group**

|  | Leptin | | Resistin | |
| --- | --- | --- | --- | --- |
|  | *r* | *P* value | *r* | *P* value |
| Age(years) | 0.022 | 0.880 | 0.258 | 0.077 |
| BMI(kg/m^2^) | 0.311 | 0.031 | -0.254 | 0.082 |
| HbA1c(%) | -0.112 | 0.449 | -0.089 | 0.549 |
| HOMA-IR | 0.189 | 0.197 | -0.198 | 0.177 |
| HOMA-B | 0.151 | 0.307 | 0.226 | 0.122 |
| ALT(U/L) | 0.179 | 0.234 | -0.188 | 0.211 |
| AST(U/L) | 0.133 | 0.377 | -0.087 | 0.564 |
| TC(mmol/L) | 0.051 | 0.734 | -0.001 | 0.995 |
| BUN(mmol/L) | -0.047 | 0.757 | 0.427 | 0.003 |
| SCr(μmol/L) | 0.021 | 0.889 | 0.419 | 0.004 |
| eGFR(mL/min/1.73m^2^) | -0.090 | 0.549 | -0.603 | <0.001 |
| Albumin-to-creatinine ratio | 0.047 | 0.797 | 0.289 | 0.108 |

**Sup Table 4 Correlations Analysis Between Adipokines Levels and Clinical Parameters in the MARD group**

|  | Leptin | | Resistin | |
| --- | --- | --- | --- | --- |
|  | *r* | *P* value | *r* | *P* value |
| Age(years) | 0.151 | 0.106 | 0.005 | 0.960 |
| BMI(kg/m^2^) | 0.433 | <0.001 | -0.009 | 0.922 |
| HbA1c(%) | 0.008 | 0.931 | 0.071 | 0.451 |
| HOMA-IR | 0.075 | 0.426 | 0.093 | 0.321 |
| HOMA-B | -0.073 | 0.436 | -0.004 | 0.966 |
| ALT(U/L) | -0.114 | 0.224 | -0.031 | 0.744 |
| AST(U/L) | -0.086 | 0.362 | -0.043 | 0.651 |
| TC(mmol/L) | 0.159 | 0.087 | 0.034 | 0.720 |
| BUN(mmol/L) | -0.033 | 0.724 | 0.140 | 0.136 |
| SCr(μmol/L) | -0.003 | 0.974 | 0.444 | <0.001 |
| eGFR(mL/min/1.73m^2^) | -0.182 | 0.052 | -0.421 | <0.001 |
| Albumin-to-creatinine ratio | -0.046 | 0.690 | 0.190 | 0.095 |
