# Supplementary material for: Roles of plasma leptin and resistin in novel subgroups of type 2 diabetes driven by cluster analysis
Source: Lipids Health Dis. 2022 Jan 7;21:7. doi: 10.1186/s12944-022-01623-z (PMC8742314; doi:10.1186/s12944-022-01623-z)
Supplement: Supplementary file 2 — Additional file 2: Supplementary Tables 5–8. Logistic regression analysis of risk factors for renal complications in the MOD, SIDD, SIRD and MARD subgroups. [file 12944_2022_1623_MOESM2_ESM.docx]

**Supplemental tables 5-8**

|  |  | OR (95%CI)^a^ | *P* ^a^ | OR (95%CI)^b^ | *P* ^b^ |
| --- | --- | --- | --- | --- | --- |
| Resistin | By median^c^ |  |  |  |  |
|  | <Median | Ref |  | Ref |  |
|  | ≥Median | 2.020(0.623-6.557) | 0.242 | 2.041(0.589-7.073) | 0.261 |
| HOMA-IR | By median |  |  |  |  |
|  | <Median | Ref |  | Ref |  |
|  | ≥Median | 1.417(0.444-4.521) | 0.556 | 1.687(0.482-5.900) | 0.413 |

**Sup Table 5 Logistic regression analysis of risk factors for renal complications in the MOD group.**

^a^ Logistic regressions was unadjusted.

^b^ Logistic regression was adjusted for sex and age at onset.

^c^ The median of all subjects were used as cutoffs when creating groups.

|  |  | OR (95%CI)^a^ | *P* ^a^ | OR (95%CI)^b^ | *P* ^b^ |
| --- | --- | --- | --- | --- | --- |
| Resistin | By median^c^ |  |  |  |  |
|  | <Median | Ref |  | Ref |  |
|  | ≥Median | 1.692(0.656-4.363) | 0.276 | 1.526(0.568-4.101) | 0.403 |
| HOMA-IR | By median |  |  |  |  |
|  | <Median | Ref |  | Ref |  |
|  | ≥Median | 1.342(0.524-3.439) | 0.540 | 1.457(0.537-3.955) | 0.460 |

**Sup Table 6 Logistic regression analysis of risk factors for renal complications in the SIDD group.**

^a^ Logistic regressions was unadjusted.

^b^ Logistic regression was adjusted for sex and age at onset.

^c^ The median of all subjects were used as cutoffs when creating groups.

**Sup Table 7 Logistic regression analysis of risk factors for renal complications in the SIRD group.**

|  |  | OR (95%CI)^a^ | *P* ^a^ | OR (95%CI)^b^ | *P* ^b^ |
| --- | --- | --- | --- | --- | --- |
| Resistin | By median^c^ |  |  |  |  |
|  | <Median | Ref |  | Ref |  |
|  | ≥Median | 4.048(1.210-13.538) | 0.023 | 6.188(1.003-12.221) | 0.049 |
| HOMA-IR | By median |  |  |  |  |
|  | <Median | Ref |  | Ref |  |
|  | ≥Median | 1.059(0.335-3.353) | 0.922 | 1.441(0.398-5.220) | 0.578 |

^a^ Logistic regressions was unadjusted.

^b^ Logistic regression was adjusted for sex and age at onset.

^c^ The median of all subjects were used as cutoffs when creating groups.

|  |  | OR (95%CI)^a^ | *P* ^a^ | OR (95%CI)^b^ | *P* ^b^ |
| --- | --- | --- | --- | --- | --- |
| Resistin | By median^c^ |  |  |  |  |
|  | <Median | Ref |  | Ref |  |
|  | ≥Median | 4.401(1.941-9.977) | <0.001 | 6.188(2.477-15.458) | <0.001 |
| HOMA-IR | By median |  |  |  |  |
|  | <Median | Ref |  | Ref |  |
|  | ≥Median | 2.419(1.115-5.247) | 0.025 | 2.269(1.024-5.028) | 0.044 |

**Sup Table 8 Logistic regression analysis of risk factors for renal complications in the MARD group.**

^a^ Logistic regressions was unadjusted.

^b^ Logistic regression was adjusted for sex and age at onset.

^c^ The median of all subjects were used as cutoffs when creating groups.
