# Supplementary figures and images for: Roles of plasma leptin and resistin in novel subgroups of type 2 diabetes driven by cluster analysis
Source: Lipids Health Dis. 2022 Jan 7;21:7. doi: 10.1186/s12944-022-01623-z (PMC8742314; doi:10.1186/s12944-022-01623-z)

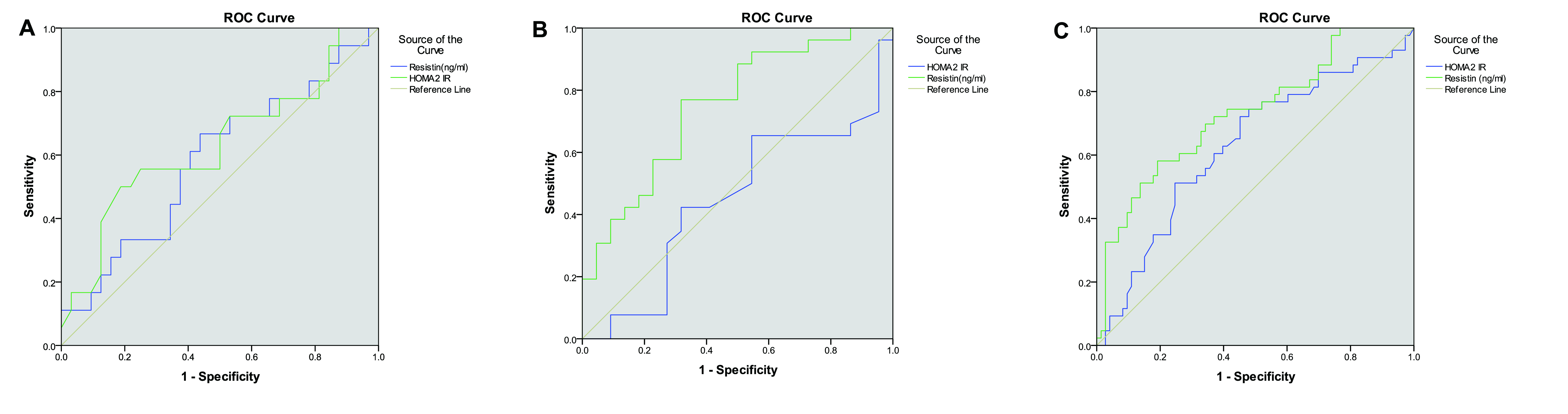

Supplement: Supplementary file 3 — Additional file 3: Supplementary Fig. 1. Receiver operating characteristic (ROC) curve analysis of resistin and HOMA2-IR for diabetic nephropathy in the other three subgroups (MOD, SIDD and MARD) [file 12944_2022_1623_MOESM3_ESM.tif]
